# Oocytes Polar Body Detection for Automatic Enucleation

**DOI:** 10.3390/mi7020027

**Published:** 2016-02-14

**Authors:** Di Chen, Mingzhu Sun, Xin Zhao

**Affiliations:** 1 Institute of Robotics and Automatic Information System (IRAIS), Nankai University, No. 94 Weijin Road, Nankai District, Tianjin 300000, China; chendi@mail.nankai.edu.cn (D.C.); zhaoxin@nankai.edu.cn (X.Z.); 2 Tianjin Key Laboratory of Intelligent Robotics (TJKLIR), Nankai University, No. 94 Weijin Road, Nankai District, Tianjin 300000, China

**Keywords:** micromanipulation, oocyte polar body detection, machine learning, polar body prediction

## Abstract

Enucleation is a crucial step in cloning. In order to achieve automatic blind enucleation, we should detect the polar body of the oocyte automatically. The conventional polar body detection approaches have low success rate or low efficiency. We propose a polar body detection method based on machine learning in this paper. On one hand, the improved Histogram of Oriented Gradient (HOG) algorithm is employed to extract features of polar body images, which will increase success rate. On the other hand, a position prediction method is put forward to narrow the search range of polar body, which will improve efficiency. Experiment results show that the success rate is 96% for various types of polar bodies. Furthermore, the method is applied to an enucleation experiment and improves the degree of automatic enucleation.

## 1. Introduction

Enucleation is a crucial step in cloning. In this step, genetic materials, including the nucleus and polar body, are removed from the oocyte [1,2,3]. Conventionally, skilled operators use two glass micropipettes to hold the oocyte and extract genetic materials manually in classical enucleation [4,5,6,7,8]. In the field of automated cell manipulation [9,10,11,12], some researchers tried to achieve enucleation automatically. Ichikawa *et al.* [13,14] used microfluidics to cut the oocyte, and then used electrical stimulation to fuse the nucleus with fertilized egg. Tatham *et al.* [15] extracted the nucleus by centrifugation due to the different densities of nucleus and cytoplasm. These methods need complex equipment and cause huge mechanical damage to oocytes. We focus on automatic blind enucleation using a micro-manipulation system.

Since the nucleus is invisible under bright-field microscope, operators get the position of the nucleus according to the position of the polar body in blind enucleation. The polar body is a small cell, which contains a small amount of cytoplasm and a copy of the genetic information of the oocyte. When a diploid cell undergoes cytokinesis after meiosis to produce the egg cell, the polar body is generated [8]. It is located between the cytoplasm and the zona pellucida, which is close to the nucleus. In blind enucleation, operators rotate the oocyte to find the polar body, and then remove it, as well as the nucleus. At present, robotic oocyte rotation has been achieved with high precision [16], but the polar body cannot be detected robustly, which leads to the failure of automatic enucleation.

**Figure 1:**
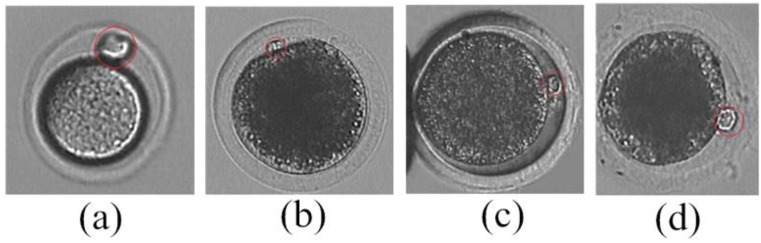
Polar body microscopic image. (**a**) The polar body of a mouse oocyte; (**b–d**) The polar bodies of porcine oocytes.

A number of methods have been reported to detect the polar body. Leung *et al.* [17] proposed a method to detect the polar body of a mouse oocyte based on image binarization. However, this method will fail if the gray level of the polar body in the microscopic image is similar to its surroundings. Wang *et al.* [18] proposed a polar body detection method based on image texture. This method obtained the best microscope light intensity for polar body detection by texture analysis, so that polar bodies of different animals can be detected. However, the lack of oocyte texture in the image and the limitation of light regulation of the microscope lead to low accuracy of the method. As shown in Figure 1, the shapes of polar bodies are quite different. The polar body of a mouse oocyte (Figure 1a) is nearly spherical, while polar bodies of porcine oocytes are hemispherical (Figure 1b–d). It is difficult to detect various polar bodies accurately and quickly.

In fact, skilled operators find a polar body in a microscopic image easily, since they identify the polar body by its shape features as well as its possible position. Similarly, we detect polar bodies based on the machine learning method in this paper. On one hand, the improved Histogram of Oriented Gradient (HOG) algorithm is employed to extract features of the polar body image, which will increase the success rate. On the other hand, a position prediction method is put forward to narrow the search range of the polar body, which will improve efficiency.

This paper is organized as follows. The polar body detection method is proposed in Section 2. Section 3 shows the experiment results of this method. In Section 4, we apply the method to an enucleation experiment. The paper is concluded in Section 5.

## 2. Learning and Prediction-Based Polar Body Detection Method

Unlike the direct detection methods [17,18], we convert this problem into image classification. A three-step position prediction–presence determination–position detection method is utilized in this paper to obtain the position of the polar body in the microscopic image. The polar body detection process and the key technologies are described below.

### 2.1. Polar Body Detection Process

**Figure 2:**
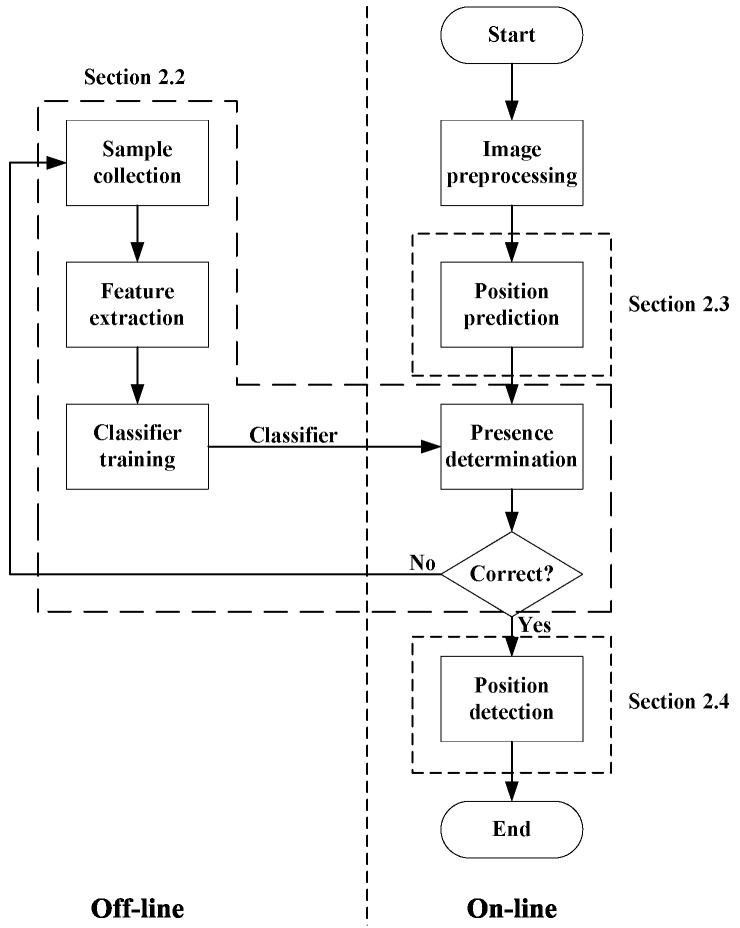
The process of polar body detection.

As shown in Figure 2, the polar body detection process is divided into two parts: the off-line part and the on-line part. The classifier of the polar body is trained in the off-line part, while the polar body in the microscopic image is detected in the on-line part.

In the off-line part, we collect image patches with or without polar bodies as positive or negative samples. Then, the improved HOG algorithm and Principal Component Analysis (PCA) algorithm [19] are used to obtain features of the polar body images. At last, the polar body classifier is trained using Support Vector Machine (SVM) algorithm [20]. We will discuss the improved HOG algorithm and SVM classification in Section 2.2.

In the on-line part, there are three main steps:

1. Obtain the image region of the oocyte as the region of interest (ROI). Divide the image region into small patches, and get the image patches that may contain the polar body by position prediction. We will analyze the method of position prediction in Section 2.3.
2. Use an SVM classifier to determine whether a polar body is present in these image patches. The detected image patches will be added into the sample library and the classifier will be trained again.
3. Detect the position of the polar body in the image patch, which is described in Section 2.4.

### 2.2. Learning-Based Polar Body Presence Determination

#### 2.2.1. Improved HOG Algorithm

As shown in Figure 1, the image gradient changes obviously in the image region of the polar body. Actually, the change of oriented gradient in the polar body region is much greater than in the non-polar body region. Therefore, we use histogram of oriented gradient to describe the features of polar body images, and calculate features in image patches with and without a polar body using the HOG algorithm [21].

**Figure 3:**
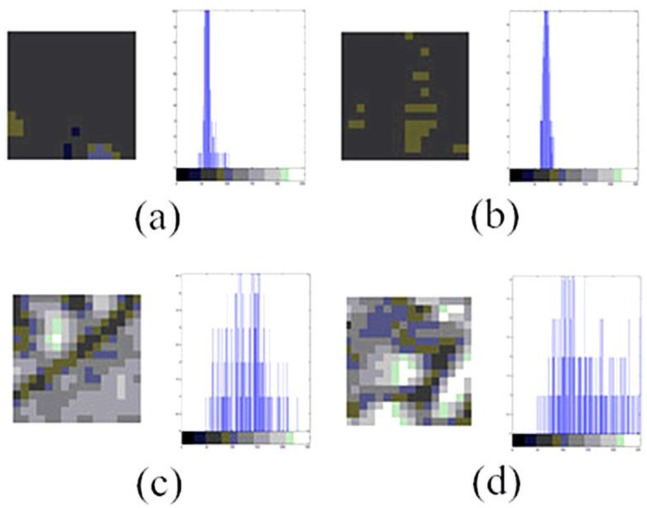
Local images and their gradient histograms in polar body and non-polar body image patches. (**a**) Image and gradient histogram around a non-polar body image patch; (**b**) Image and gradient histogram around a polar body image patch; (**c**) Image and gradient histogram in the center of a non-polar body image patch; (**d**) Image and gradient histogram in the center of a polar body image patch.

Generally, polar bodies are set in the center of polar body image patches. We further analyze the different image regions in image patches. Figure 3 shows the local images, as well as their gradient histograms, around and in the center of image patches. The gradient histograms of local images around polar body and non-polar body image patches are nearly the same, while the gradient histograms of the center parts are quite different.

**Figure 4:**
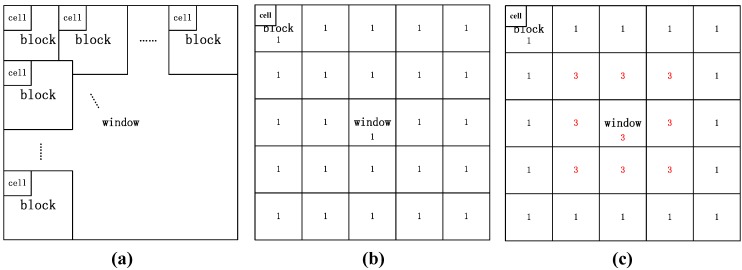
Original Histogram of Oriented Gradient (HOG) algorithm and improved HOG algorithm. (**a**) Original HOG algorithm; (**b**) Weights of blocks in the original HOG algorithm; (**c**) Weights of blocks in the improved HOG algorithm.

In the HOG algorithm, the image is divided into window, blocks, and cells, as shown in Figure 4a. HOG is calculated in each cell and combined to construct HOG of blocks and window. As shown in Figure 4b, the weights of blocks in different parts of window are the same in the original HOG algorithm [21].

We improve the HOG algorithm for polar body images by setting different weights to blocks in different positions. Figure 4c shows the weight matrix, where the weights of nine blocks in the center are set as three, while weights of other blocks are set as one. Experimental results show that the success rate increases by 5% due to this improvement.

#### 2.2.2. Polar Body Presence Determination Based on SVM Algorithm

In this paper, SVM algorithm [20] is used to classify polar body and non-polar body image patches. The main steps of polar body presence determination are listed as follows:

1. Extract features of polar body and non-polar body image patches (positive and negative samples) using improved HOG algorithm.
2. Reduce the dimension of features using PCA algorithm [19].
3. Train the polar body classifier using SVM algorithm.
4. Extract features of the detected image patch and determine whether a polar body is present in it using the polar body classifier.

In order to make the classifier more accurate, the sample library for SVM training is increased dynamically in this paper. The detected image patches are added automatically into the sample library. The classifier is trained again when the number of samples changes.

### 2.3. Polar Body Position Prediction

**Figure 5:**
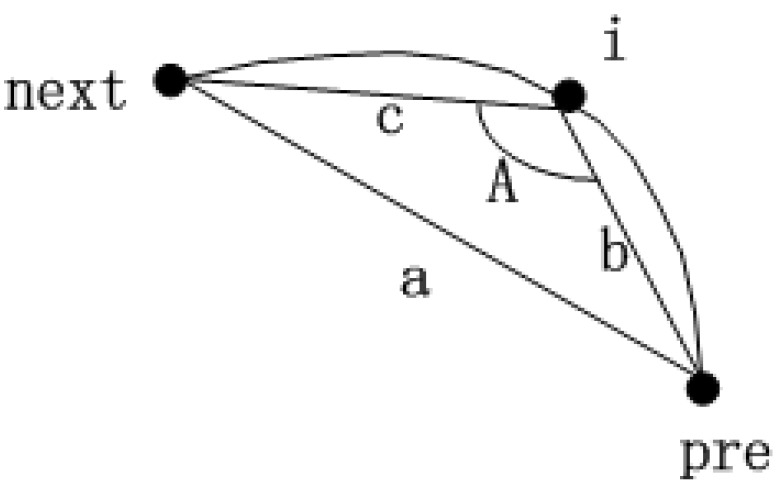
Schematic diagram of cosine algorithm.

In this paper, polar body position prediction is used to shorten detection time. There are two steps in polar body position prediction:

1. Obtain cytoplasmic membrane contour. The polar body is located between the cytoplasm and the zona pellucida. We use the cytoplasmic membrane contour to determine the candidate positions of the polar body. The main steps in obtaining the cytoplasmic membrane contour are listed as follows:
  (1) The de-noised image is processed by using intensity transformation, so that the zona pellucida is eliminated in the image. The region of cytoplasmic membrane is detected by using binarization.
  (2) The region of cytoplasm is further detected by using Hoff algorithm [22].
  (3) The edge information is acquired by using Canny algorithm [23].
  (4) The cytoplasmic membrane contour is detected by using the active contour method.
2. Analyze the curvature of cytoplasm membrane contour to narrow the detection range of polar body. As shown in Figure 1, the curvature of cytoplasm membrane contour in polar body position is obviously different with the non-polar body position. The main steps are listed as follows:
  (1) For each polar body candidate position, we select two neighbor points in the contour with same interval. These three contour points compose a triangle. Figure 5 shows the schematic diagram, where Point i represents the candidate position, Point pre and Point next are the neighbor points.
  (2) We calculate the cosine value of angle A instead of the curvature.
  (3) The cosine value is smaller if Point i is in the position of the polar body; We set a threshold to distinguish the position of polar body and non-polar body. The threshold is obtained experimentally.

### 2.4. Polar Body Position Detection

The image patches are extracted according to the results of polar body position prediction. Polar body position detection is used to obtain the coordinates of the polar body in the microscopic image. The main steps of obtaining the coordinates of the polar body are listed as follows:

1. The polar body is detected by using SVM classifier in the image patch.
2. The contour of the polar body is detected by using image binarization.
3. The polar body position is obtained by calculating the center of the polar body contour in the binarization result.

## 3. Experiments

### 3.1. Sample Library Establishment

The sample library contains a large number of image patches with or without a polar body as positive or negative samples. The size of the image patches, which depends on the resolution of the polar body image, is set to 64 × 64 in this paper. The samples are important for detection. We build the sample library according to these principles:

1. We should consider detection efficiency when choosing samples.
2. There should be obvious differences between positive samples and negative samples.
3. There should be various types of polar bodies in positive samples.

**Figure 6:**
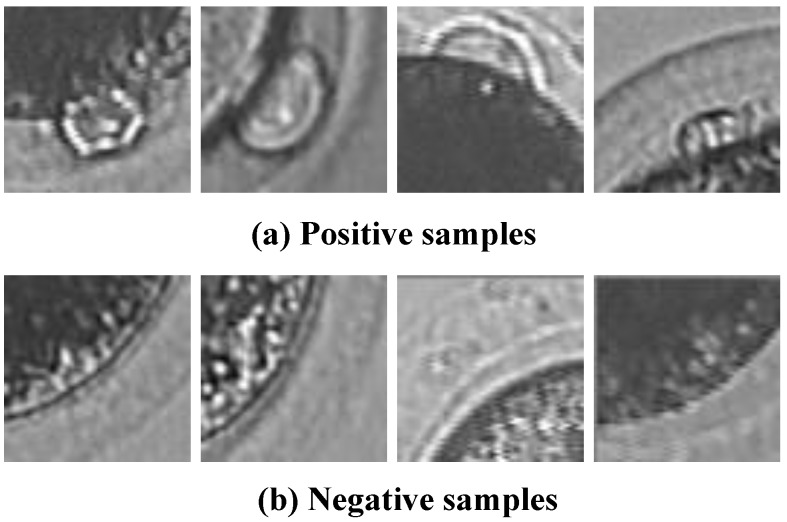
The sample library. (**a**) Polar body-Positive samples; (**b**) Polar body-Negative samples.

Initially, there are 1200 positive samples and 2000 negative samples in sample library. Figure 6 shows some typical positive and negative samples.

### 3.2. HOG Feature Parameters Selection

The improved HOG algorithm [21] is used to extract the feature of the polar body. The selection of HOG parameters is very important for the success rate. The HOG parameters, which include cell size, block size, and step length of the block, are analyzed by using 72 images.

1. Block size.
  We change block size with the same feature dimension. The test results are shown in Table 1, which indicate that the block size has little effect on detection results.

**Table 1:** The test result of block size.

Block Size	Correct Number	Wrong Number	Success Rate
32 × 32	67	5	93%
24 × 24	67	5	93%
16 × 16	67	5	93%

2. Cell size in a block.
  We change cell size in a block with the same feature dimension. The test results are shown in Table 2, which indicate that the success rate increases with large cell size in a block.

**Table 2:** The test result of the number of cells in a block.

Cell Number	Correct Number	Wrong Number	Success Rate
4	67	5	93%
9	69	3	95.8%

3. Step length and the size of overlapping.
  In the process of block moving, an overlapped block may produce interference information, but it can also provide a better detection in classifier [21]. We change step length with the same feature dimension. The test results are shown in Table 3.

**Table 3:** Block step length test result.

Block Step Length	Correct Number	Wrong Number	Success Rate
20	58	14	81%
10	61	11	85%
8	62	10	86%

In conclusion, the HOG parameters in our method are set as follows: Window size (the size of image patches) is 64 × 64, block size is 24 × 24, and cell size is 8 × 8. The step length of the block is 10. The feature dimension is 2025.

### 3.3. PCA Feature Parameters Selection

PCA algorithm [19] is used to reduce feature dimension. The dimension is chosen as 10, 20, 30, and 40 in experiments, The comparison results are shown in Table 4, which indicate that the proper dimension is 30.

**Table 4:** PCA test results.

Dimension	Correct Number	Wrong Number	Success Rate	Time (ms/Frame)
10	47	25	34.7%	115
20	61	11	84.7%	123
30	69	3	95.8%	130
40	69	3	95.8%	147
2025	70	2	97.2%	605

### 3.4. Polar Body Position Prediction Results

**Figure 7:**
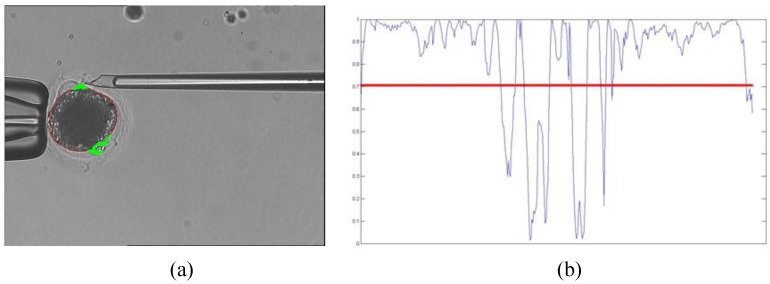
Polar body position prediction result. (**a**) The prediction result; (**b**) The cosine value.

We use the prediction method to obtain polar body candidate positions. The prediction result is shown in Figure 7. In Figure 7a, the thick green line represents the polar body candidate positions, while the corresponding cosine values in the contour of the cytoplasmic membrane are shown in Figure 7b. The threshold is set to 0.707 in this paper. When the cosine values are less than the threshold, the contour points are set as candidate positions.

**Figure 8:**
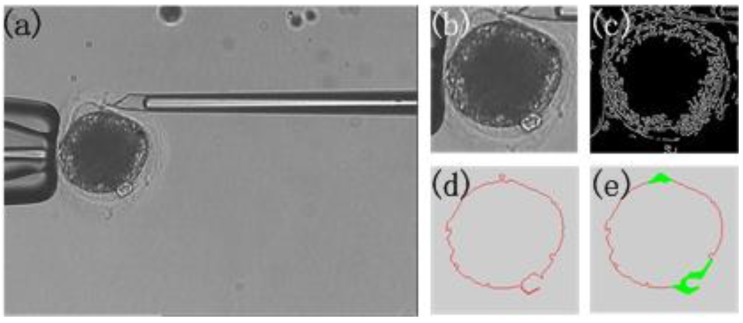
Prediction result. (**a**) Microscopic image; (**b**) The oocyte area; (**c**) Edge detection result; (**d**) The contour of the oocyte; (**e**) The prediction result.

The result of each step of the prediction is shown in Figure 8. The detection time is shortened by using the position prediction.

### 3.5. Polar Body Detection Results

**Figure 9:**
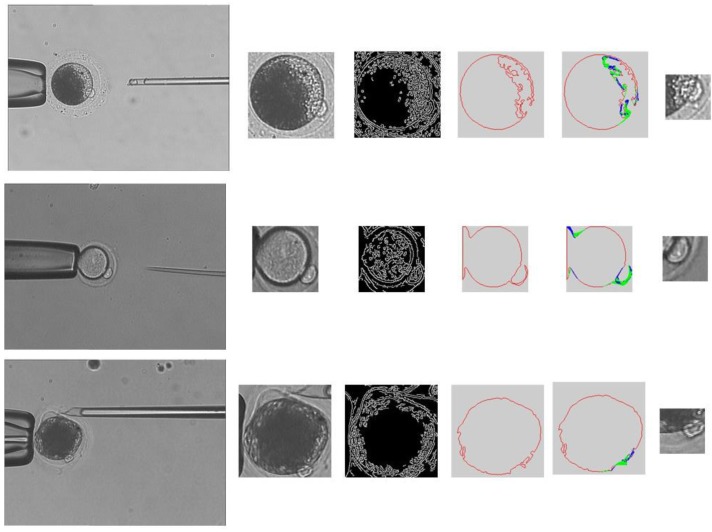
Detection results for various oocytes.

We run the polar body detection algorithm in the computer (Intel I3 processor, 2G RAM) with Visual Studio 2010 and OpenCV. The OpenCV version 2.4 and C++ interface are chosen for our method. We tested 8722 images in total. Figure 9 shows some typical detection results. In Figure 9, the polar bodies of porcine oocytes and mouse oocytes are detected by our method. The shapes and appearances of polar bodies in these oocytes are quite different. Original microscopic images, region of interest (ROI), edge detection results, contours of cytoplasmic membrane, and prediction results are shown from left to right.

The statistical results of polar body detection are shown in Table 5. The detection time of one image is about 130 ms.

**Table 5:** The result of the positioning polar body.

	TP	TN	FP	FN	Success Rate
Number	2277	6131	107	107	96.4%

This method is compared with the previous methods [17,18]. We consider three indices: success rate, availability when oocyte is rotated, and range of application. The comparison results are shown in Table 6. Success rate of our method is high and the robustness is improved.

**Table 6:** Comparison of methods.

Method	Success Rate	Availability When Oocyte Is Rotated	Range of Application
Detection based on the binarization [17]	-	No	Mouse oocytes
Detection based on the texture [18]	80%	No	Many types of cells
Our method	96%	Yes	Many types of cells

## 4. Application of Polar Body Detection Method in Automated Enucleation

There are three steps in the blind enucleation process [16]:

(1) Hold the oocyte using a holder micropipette.
(2) Rotate the oocyte using an injection micropipette until the polar body is in the proper position.
(3) Localize the polar body and remove the nucleus and polar body using an injection micropipette.

**Figure 10:**
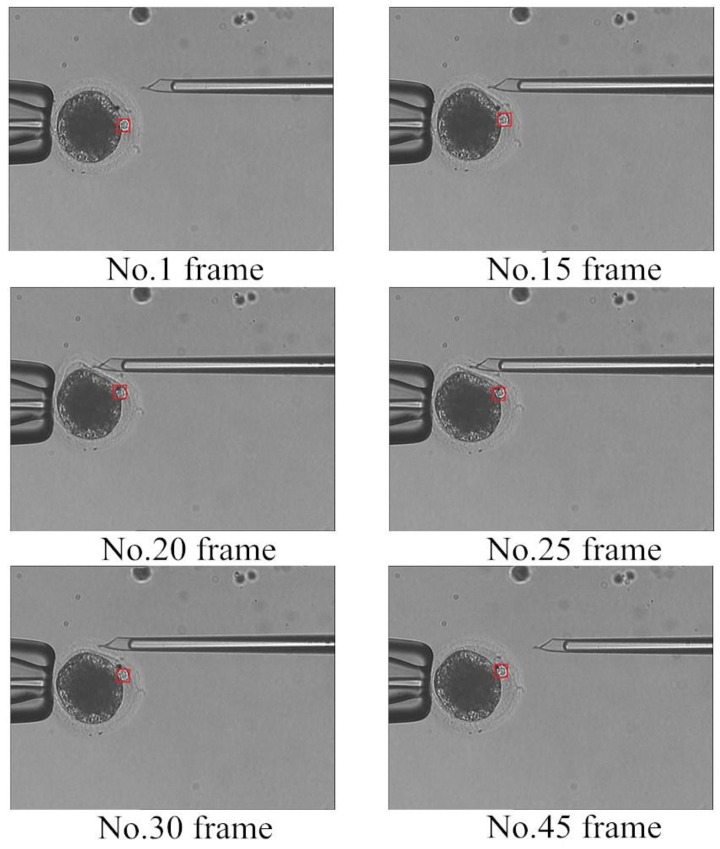
Detection results of oocyte rotation.

The polar body detection method is applied to improve the automatic degree of enucleation in steps (2) and (3). In step (2), the oocyte is rotated, the detection method is used to determine whether the polar body is present, and the polar body moves to a suitable position for removal. Figure 10 shows the detection results when the oocyte is rotated, where a red rectangle represents the detection position of the polar body.

**Figure 11:**
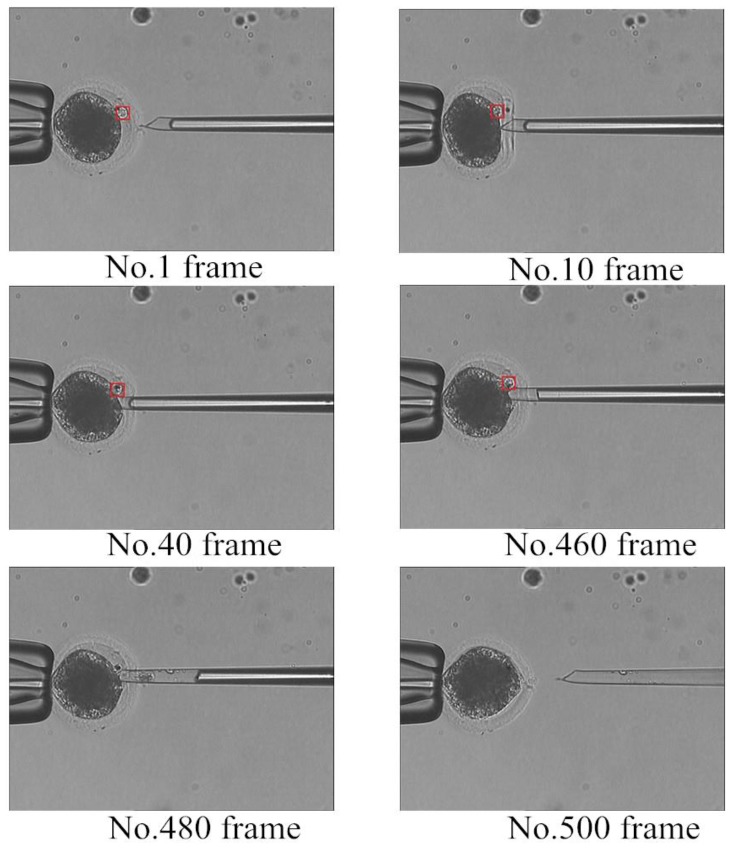
Detection results of the enucleation process.

In step (3), the genetic material is extracted and the polar body detection method is used to determine whether the polar body exists. Figure 11 shows the enucleation process. In frame No. 480, the polar body disappears and the enucleation process ends. It is the time to take micropipette out.

## 5. Conclusions

Low success rate and low efficiency in polar body detection blocks automatic enucleation. In this paper, a learning-based detection method is proposed to solve this problem. The improved HOG algorithm and the position prediction method are employed to improve success rates and efficiency, respectively. Experimental results show that the method can detect many types of polar bodies in oocytes. The success rate is 96% for 8722 test images, and the detection time of one image is about 130 ms. Furthermore, the method is applied to an automatic enucleation experiment to detect the position of the polar body, as well as to determine whether the polar body disappears. The experimental results indicate that the method improves the automatic degree of enucleation successfully. In the future, we will combine the polar body detection method, the oocyte rotation method, and the control method of micro-injector to achieve automatic enucleation.
